# Ketogenic diet prevents chronic sleep deprivation-induced Alzheimer’s disease by inhibiting iron dyshomeostasis and promoting repair *via* Sirt1/Nrf2 pathway

**DOI:** 10.3389/fnagi.2022.998292

**Published:** 2022-09-01

**Authors:** Yueqi Yang, Xueyan Wang, Aiai Xiao, Jun Han, Zhengping Wang, Min Wen

**Affiliations:** Institute of Biopharmaceutical Research, Liaocheng University, Liaocheng, China

**Keywords:** chronic sleep deprivation, Alzheimer’s disease, ketogenic diet (KD), ferroptosis, SIRT1, Nrf2

## Abstract

Sleep deprivation (SD) is one of the main risk factors for Alzheimer’s disease (AD), but the underlying mechanism is still unclear. Ketogenic diet (KD) has been shown widely neuroprotective effects but less known about its effect on SD-induced AD. In the present study, a continuous 21 days SD mouse model with or without KD was established. The changes of cognitive function, pathological hallmarks of AD, ferroptosis, and intracellular signal pathways in mice were detected by Morris water maze, ThS staining, diaminobenzidine (DAB)-enhanced Perls’ stain, antioxidant assay, immuno-histochemistry, and western blot. The results showed that KD can prevent the cognitive deficiency, amyloid deposition and hyperphosphorylated tau induced by chronic SD. Analysis of ferroptosis revealed that KD can inhibit iron dyshomeostasis by down-regulating the expression of TfR1 and DMT1 and up-regulating the expression of FTH1, FPN1. Meanwhile, KD alleviated oxidative stress with elevated xCT/GPX4 axis, FSP1 and reduced MDA. In addition, KD could promote neuronal repair by enhancing BDNF and DCX. Further studies demonstrated that KD activated Sirt1/Nrf2 signaling pathway in the hippocampus in SD-exposed mice. Our finding firstly suggested that KD could prevent chronic SD-induced AD by inhibiting ferroptosis and improving the neuronal repair ability *via* Sirt1/Nrf2 signaling pathway.

## Graphical Abstract

KD could prevent chronic SD-induced AD by inhibiting ferroptosis *via* Sirt1/Nrf2 signaling pathway. 
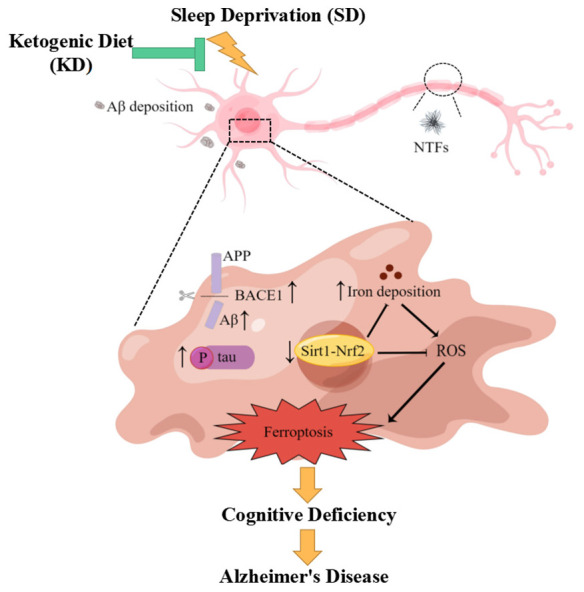

## Introduction

Alzheimer’s disease (AD) is a common neurodegenerative disease and characterized by β-amyloid (Aβ) deposition, neurofibrillary tangles resulted from hyperphosphorylated tau, and neuronal loss (Ashraf and So, 2020). At present, although the amount of AD prevalence is growing year by year, there is no effective drug for AD, so it is vital to prevent AD. AD can be divided into familial and sporadic AD which accounts for more than 95% of AD cases and is caused by the combination of aging and environmental factors, including sleep disorder (Abulafia et al., 2017). With the change of lifestyle, sleep deprivation (SD) has become a common phenomenon in modern society and is recognized as one of the main risk factors for AD (Van Erum et al., 2018). There is a bidirectional link between SD and AD. AD patients are normally accompanied by different sleep problems, such as trouble falling asleep, and sleep interruptions. At the same time, long-term SD can lead to Aβ deposition, abnormally phosphorylated tau, which causes hippocampal damage and cognitive impairment, and thereby promoting the occurrence of AD (Han et al., 2021). Nevertheless, the underlying molecular mechanism of SD on AD remains largely unclear. Investigating the mechanism of SD-induced AD and executing targeted interventions may have significant implications for early prevention of AD.

Iron is the most abundant metal in the brain and is involved in multiple physiological processes to maintain normal brain function. Generally, the level of iron in the brain is slightly increased with aging, but iron deposition is more significant in the brains of AD patients (Yan and Zhang, 2019). The iron concentration in the hippocampus correlates positively with the Aβ deposition, but inversely with cognitive performance of AD patients (Yan and Zhang, 2019). Using iron chelator (deferoxamine) could reduce the level of iron in the brain and suppress the progression of AD, indicating an important role of iron dyshomeostasis in AD (Crowe and Morgan, 1994; Gassen and Youdim, 1997). The excessive iron can increase ROS and induce the production of lipids peroxide, ultimately leading to ferroptosis. Ferroptosis is an iron-dependent form of programed non-apoptotic cell death, characterized by intracellular iron deposition and accumulation of lipid peroxide products. Current research indicates that ferroptosis is linked to a variety of neurodegenerative diseases, including AD (Reichert et al., 2020). In addition, emerging evidence has reported that SD-caused memory impairment is related to hippocampal ferroptosis and can be reversed by ferrostatin-1, a specific inhibitor of ferroptosis (Wang X. et al., 2021, 2022). The above studies show that ferroptosis can act as a bridge between SD and AD, and targeting ferroptosis may be an effective means of preventing AD.

The ketogenic diet (KD) is characterized by high-fat, low-carbohydrate, which result in elevated levels of ketones, mainly β-hydroxybutyrate (BHB) in the blood (Ricci et al., 2020). Generally, the ratio of total energy from fat to carbohydrate and protein combined of KD is from 1:1 to 4:1, which reflects the ketogenic level and strictness of KD (Ricci et al., 2020). KD has aroused wide public concern owing to its therapeutic application in epilepsy successfully. Studies have uncovered that KD exerts neuroprotective effects against neurodegenerative diseases including AD, Parkinson’s diseases (PD) and cognitive impairment (Pavón et al., 2021). Currently, studies have shown that the neuroprotective effects of KD involve antioxidant, anti-inflammatory and energy metabolism (Hernandez et al., 2018). Our latest research suggests that KD could ameliorate chronic SD-induced cognitive impairment by inhibiting hippocampus neuron ferroptosis (Wang X. et al., 2022), this indicates to us that KD could be used as a potential dietary treatment for the prevention of AD caused by SD. However, it is unclear whether and how KD treatment could suppress the occurrence of AD induced by chronic SD.

Accordingly, the present study intended to assess (1) the prophylactic effect of KD on the development of AD induced by chronic SD, (2) the effect of KD on SD-induced hippocampal damage, and (3) the molecular mechanisms by which KD mediates in preventing SD-induced AD.

## Materials and methods

### Reagents and antibodies

Bicinchoninic acid (BCA) protein assay kit was purchased from Beyotime Biotechnology (Shanghai, China). Malondialdehyde (MDA) and glutathione (GSH) assay kit were purchased from Nanjing Jiancheng Bioengineering Institute (Jiangsu, China). Beta-actin (β-actin), beta-myloid precursor protein cleavage enzyme-1 (BACE1), amyloid precursor protein (APP), amyloid β-protein (Aβ), microtubule-associated protein tau (tau), phosphorylated-tau (*p*-tau), brain-derived neurotrophic factor (BDNF), doublecortin (DCX), iron regulatory proteins 1 (IRP1), iron regulatory proteins 2 (IRP2), transferrin receptor 1 (TfR1), divalent metal-ion transporter-1 (DMT1), ferritin heavy chain 1 (FTH1), ferroportin 1 (FPN1), glutathione peroxidase 4 (GPX4), system xc-cystine-glutamate antiporter (xCT), ferroptosis suppressor protein 1 (FSP1), sirtunin 1 (Sirt1), nuclear factor E2 related factor 2 (Nrf2), phosphorylated-Nrf2 (*p*-Nrf2), and secondary HRP-conjugated antibodies were purchased from Abcam (Cambridge, MA, United States).

### Animals and treatments

**FIGURE 1:**
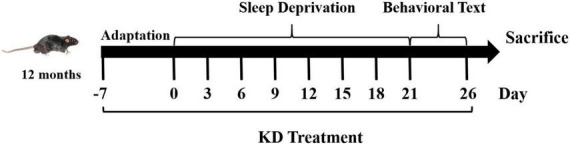
Flow schematic depiction of study design.

Consider that the risk of AD is higher in women than in men (Smith et al., 2020), a total of thirty female C57BL/6 mice (12-month-old) were used in this study. Animals were kept on a 12-h light/dark cycle at room temperature of 22 ± 2°C and received water and food *ad libitum*. All animal procedures were approved by the Experimental Animal Ethics Committee of Liaocheng University, and were done in accordance with the National Institutes of Health Guide for the Care and Use of Laboratory Animals (NIH publications No. 80-23). Mice were randomly allocated to control group (Con) or sleep deprivation group fed with AIN93M diet (SD) or KD. The composition of the experimental diets is listed in Supplementary Table 1. Conduct behavioral tests after sleep deprivation and after all behavioral tests, the mice were sacrificed for further analysis (As shown in Figure 1).

### Chronic sleep-deprivation protocol

The model of chronic sleep deprivation was induced as we described previously (Wang X. et al., 2022). Briefly, an Automated Sleep Deprivation System (Shanghai XinRuan Information Technology CO., Ltd. Shanghai, China) was used for the chronic SD in mice. The system stops mice from sleeping by randomly rotating bars. Five mice from the same group were placed in the system and got food and water *ad libitum*. Mice were acclimated to the SD system for 7 days to minimize stress before starting the study as previously described (Zhao et al., 2019). SD was achieved with rotation (5 rev/min) from 14:00 to 10:00 + 1 day for 20 h. After sleep deprivation, mice were permitted to sleep at 10:00 and maintain for 4 h. The Con group mice stay in the same system, but are allowed normal sleep at the corresponding time.

### Morris water maze test

The Morris water maze test was executed in a circular dark pool (height 50 cm, diameter 180 cm) filled with white water (25 ± 1°C). Mice were required to find a hidden platform which was placed about 1 cm under water surface in the center of one quadrant of the pool. The mice from each group experienced four consecutive daily training trials. A probe trial was then repeated in the absence of the platform. Behaviors of the mice were tracked using Any-maze software (Stoelting Co.), the escape latency during the training period, the time in the target quadrant, and the across numbers of the target area during the probe trial were analyzed.

### Biochemical analysis

After behavioral experiments, mice were killed by inhalation of 2% isoflurane. Eyeball blood from mice in each group was collected for blood glucose and BHB and measured with handheld glucometer and ketone meter respectively (Abbott Labs, Abbott Park, IL, United States). Hippocampus were quickly isolated from the brain and frozen in liquid nitrogen immediately. The levels of total iron, MDA, and GSH were tested using kits according to the manufacturer’s protocols.

### Histopathological examination

#### Diaminobenzidine-enhanced Perls’ staining

The brain tissue was embedded in paraffin and blocks were sectioned at 5 mm thickness. Slides were immersed in 4% ferrocyanide/4% hydrochloric acid in the dark for 30 min, then incubated with diaminobenzidine (DAB) for enhanced iron staining for 30 min, and counterstained with hematoxylin subsequently. After routine dehydration and transparent treatment, the neutral gum was sealed and recorded under a bright-field microscope (Olympus, Tokyo, Japan).

#### H&E staining

Incubate the slides with hematoxylin solution in a staining jar for 5–10 min to stain the nuclei, then wash with distilled water and stained with eosin solution for 1–3 min. The sections of brain stained with H&E were observed and recorded under a bright-field microscope (Olympus, Tokyo, Japan).

#### Thioflavin-S staining

For thioflavin-S (ThS) staining, ethanol gradient-treated sections were stained with 1% ThS for 5 min and then destained with 70% ethanol. All of the sections were observed using a fluorescence microscope (Olympus, Tokyo, Japan).

#### Immunohistochemical assay

Immunohistochemical studies were executed as the previous method (Wang X. et al., 2022). Sections treated with primary antibodies against Sirt1 and DCX respectively overnight at 4°C. Next, we were treated with horseradish peroxidase-conjugated secondary antibodies for 3 h at 4°C. All of the sections were observed using a fluorescence microscope (Olympus, Tokyo, Japan).

### Western blot assay

Total proteins of hippocampus were prepared for western blotting as described in our preceding study (Wen et al., 2021). The antibodies used in this study were β-actin, BACE1, APP, Aβ, tau, P-tau, BDNF, DCX, IRP1, IRP2, TfR1, DMT1, FTH1, FPN1, GPX4, xCT, FSP1, Sirt1, Nrf2, p-Nrf2, and specific peroxidase (HRP)-conjugated secondary antibodies. These proteins were visualized using the enhanced chemiluminescence substrate and analyzed by UVP Auto Chemi Image system (Tanon 4600SF, Tanon, Shanghai, China).

### Statistical analysis

Data were expressed as mean ± standard error of the mean (mean ± SEM). The significance of mean values among different groups was analyzed by a one-way ANOVA with a Tukey’s *post hoc* test. The level of significance was considered at *p* < 0.05.

## Results

### Effects of ketogenic diet on blood β-hydroxybutyrate and blood glucose in the chronic sleep deprivation exposed mice

As expected, blood BHB levels were obviously higher in the KD group mice than in the Con group and SD group (*p* < 0.01, Supplementary Figure 1A). While, there was no significant difference in the blood glucose among these groups (Supplementary Figure 1B).

### Effects of ketogenic diet on chronic sleep deprivation-induced cognitive deficiency

**FIGURE 2:**
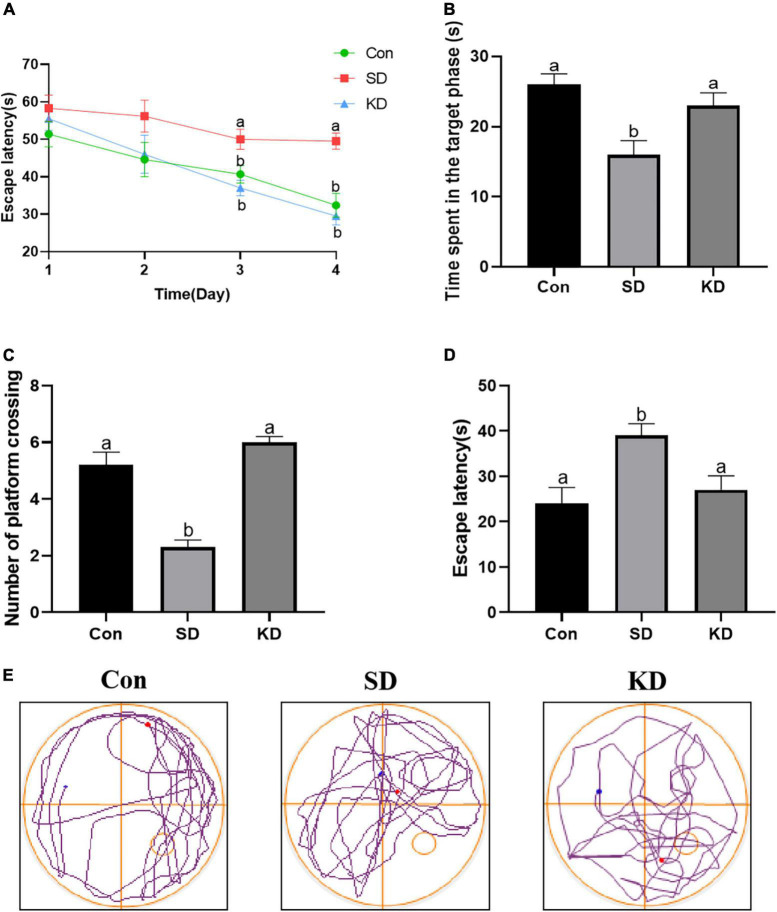
Effects of ketogenic diet (KD) on chronic sleep deprivation (SD)-induced cognitive deficiency. **(A)** The escape latency on training phase, **(B)** Time spent in the target quadrant, **(C)** Numbers of crossing platform, **(D)** escape latency, **(E)** Representative Morris water maze movement track from all groups. Data are presented as mean ± SEM (*n* = 10). Different letter indicates significantly different between each group (*p* < 0.05).

We can see from Figure 2 that the escape latency was obviously increased in the SD group compared with the Con group on the third and fourth day (*p* < 0.05). While KD treatment significantly shortened the escape latency in SD-exposed mice. Next, we evaluated the spatial memory ability of mice by a spatial probe test after finishing the navigation test. A longer periods of time finding the platform and reduced time spent in the target quadrant with less number of platform crossings were exhibited in the SD group compared to the Con group (Figures 2B–D, *p* < 0.05). Nonetheless, KD treatment could largely improve this condition in SD-exposed mice. Figure 2E shows the typical track of three groups in the probe trials. These results reflected the improved effect of KD on chronic SD-induced cognitive deficiency in mice.

### Effects of ketogenic diet on amyloid deposition and tau phosphorylation in the chronic sleep deprivation exposed mice

**FIGURE 3:**
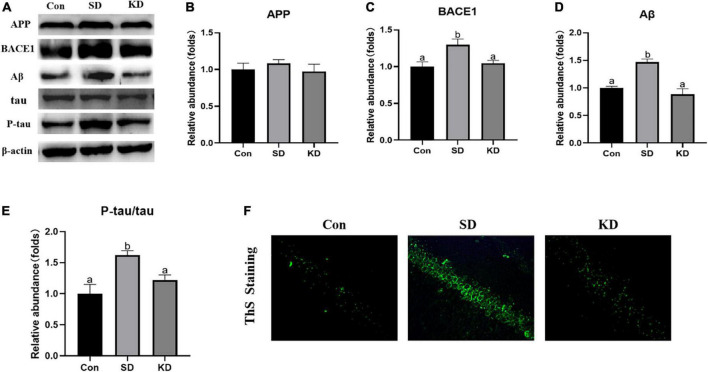
Effects of ketogenic diet (KD) on Aβ deposition and tau phosphorylation in the chronic sleep deprivation (SD) exposed mice. **(A)** Western-blots and **(B–E)** densitometry of APP, BACE1, Aβ, and p-tau/tau. Protein levels are normalized to β-actin which served as loading control and reproduced with Sham group. Values are indicated as the mean ± SEM (*n* = 7). Different letter indicates significantly different between each group (*p* < 0.05). **(F)** ThS staining in the hippocampus, scale bars = 50 μm (*n* = 3).

Amyloid deposition and neurofibrillary tangles are two typical pathological features of AD. Therefore, we first assessed the effect of KD on them to investigate the preventive effect of KD on SD-induced AD. As shown in Figure 3A, sleep deprivation for 21 days obviously induced the increase in the protein expression of BACE1, Aβ (Figures 3C,D, *P* < 0.05), and amyloid deposits (ThS Staining) (Figure 3F). No changes were noticeable in the expression of APP (Figures 3A,B). Meanwhile, SD elevated the levels of p-tau and the ratio of p-tau/tau in the hippocampus (Figures 3A,E, *P* < 0.05). Nevertheless, their levels were suppressed by KD supplementation.

### Effects of ketogenic diet on hippocampal damage in the chronic sleep deprivation exposed mice

**FIGURE 4:**
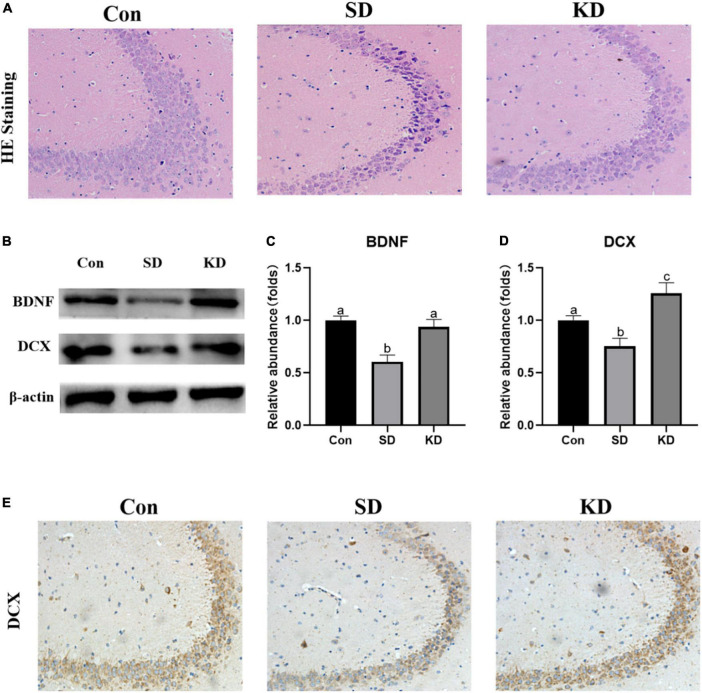
Effects of ketogenic diet (KD) on the hippocampal damage in the chronic sleep deprivation (SD) exposed mice. **(A)** H&E staining images of the hippocampus, scale bars = 50 μm (*n* = 3). **(B)** Western-blots and **(C,D)** densitometry of BDNF and DCX. Protein levels are normalized to β-actin which served as loading control and reproduced with Sham group. Values are indicated as the mean ± SEM (*n* = 7). Different letter indicates significantly different between each group (*p* < 0.05). **(E)** Immunohistochemistry analysis of DCX, scale bars = 50 μm (*n* = 3).

H&E staining showed altered neurons characterized by obviously neuronal loss and increased shrinking neurons with nuclei shrinkage in the SD group as compared with the Con group (Figure 4A). In the KD group, more clear nuclei and complete morphology of neurons were exhibited. Then we investigated the effect of KD on the repair ability of the hippocampus. The expression of neurotrophic factors BDNF and new neuron marker DCX was evaluated by western blot (Figures 4B–D). We found that both DCX and BDNF were declined in the SD group as compared with to Con group, which can be reversed by KD treatment (Figures 4B–D, *p* < 0.05). Simultaneously, the immunohistochemical results of DCX were comparable to the western blot results (Figure 4E).

### Effects of ketogenic diet on hippocampal iron dyshomeostasis in the chronic sleep deprivation exposed mice

**FIGURE 5:**
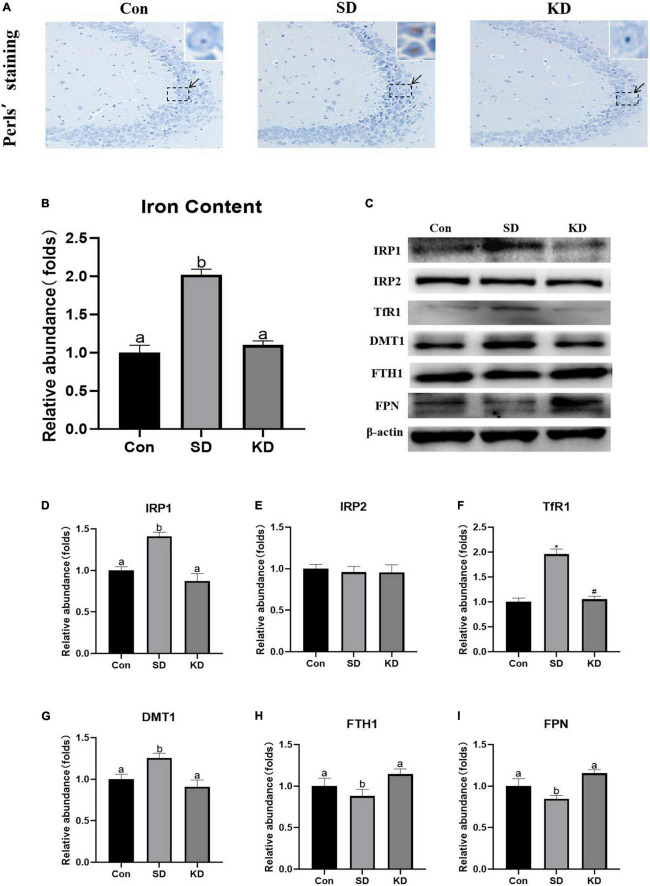
Effects of ketogenic diet (KD) on iron homeostasis dysregulation in hippocampus induced by chronic sleep deprivation (SD). **(A)** DAB staining in the hippocampus, scale bars = 50 μm (*n* = 3). **(B)** Total iron content in the brain and **(C)** Western-blots and **(D–I)** densitometry of IRP1, IRP2, TfR1, DMT1, FTH1, and FPN1. Protein levels are normalized to β-actin which served as loading control and reproduced with Sham group. Values are indicated as the mean ± SEM (*n* = 7). Different letter indicates significantly different between each group (*p* < 0.05); *p < 0.01, compared with Con; ^#^*p* < 0.01, compared with SD.

Iron accumulation is one of the main characters of ferroptosis. To evaluate the effect of KD on SD-induced iron dyshomeostasis in the hippocampus, total iron, iron accumulation and the expression of iron transport proteins were measured in the present study. We found that obvious iron deposition (Figure 5A) and increased total iron content (Figure 5B, *P* < 0.05) were presented in the SD group compared to the Con group. Meanwhile, significantly increased IRP1 (Figures 5C,D, *P* < 0.05), TfR1 (Figure 5F, *P* < 0.01) and DMT1 (Figure 5G, *P* < 0.05) accompanied with decreased FTH1 and FPN1 were shown in the SD group (Figures 5H,I, *P* < 0.05). No significant alteration was observed in IRP2 among three groups (Figure 5E). However, KD treatment reversed the expression of these iron proteins and alleviated iron deposition. These results revealed that KD supplementation could improve iron deposition by modulating the balance of iron transporter proteins.

### Effects of ketogenic diet on lipid peroxidation in the hippocampus induced by chronic sleep deprivation

**FIGURE 6:**
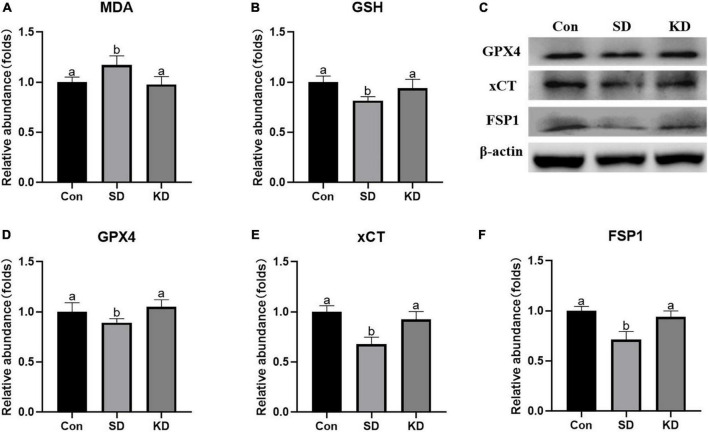
Effects of ketogenic diet (KD) on lipid peroxidation mediated oxidative stress in hippocampus induced by chronic sleep deprivation (SD). **(A)** Levels of malondialdehyde (MDA) and **(B)** glutathione (GSH). **(C)** Western-blots and **(D–F)** densitometry of GPX4, xCT, and FSP1. Protein levels are normalized to β-actin which served as loading control and reproduced with Sham group. Values are indicated as the mean ± SEM (*n* = 7). Different letter indicates significantly different between each group (*p* < 0.05).

Lipid peroxides accumulation is another characteristic of ferroptosis. Therefore, we next to investigate the effect of KD on the lipid peroxidation in the hippocampus, we determined the levels of GSH, GPX4, xCT, FSP1, and MDA. In the SD group, an elevation in MDA was noted as compared to the Con group (Figure 6A, *P* < 0.05). On the contrary, as for antioxidant enzymes, significant decrease in GSH, GPX4, xCT, and FSP1 were presented (Figures 6B–F, *p* < 0.05). However, all above alterations can be reversed by KD intervention.

### Effects of ketogenic diet on hippocampal Sirt1/Nrf2 signaling pathway in chronic sleep deprivation exposed mice

**FIGURE 7:**
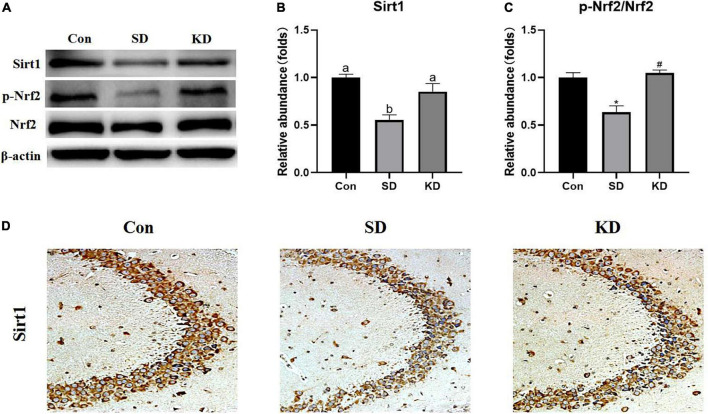
Effects of ketogenic diet (KD) on Sirt1/Nrf2 signaling pathway in hippocampus induced by chronic sleep deprivation (SD). **(A)** Western-blots and **(B,C)** densitometry of Sirt1 and p-Nrf2/Nrf2. Protein levels are normalized toβ-actin which served as loading control and reproduced with Sham group. Values are indicated as the mean ± SEM (*n* = 7). Different letter indicates significantly different between each group (*p* < 0.05); *p < 0.01, compared with Con; ^#^*p* < 0.01, compared with SD. **(D)** Immunohistochemistry analysis of Sirt1, scale bars = 50 μm (*n* = 3).

To evaluate the molecular mechanism by which KD exerted preventive effects on SD-induced AD, we examined the expression of Sirt1 and Nrf2 in the hippocampus by western blot (Figure 7A). Compared with the Con group, a down-regulation of Sirt1 (Figure 7B, *P* < 0.05), and *p*-Nrf2/Nrf2 (Figure 7C, *P* < 0.01) was exhibited in the SD groups. While KD treatment counteracted this SD-induced reduction. The results of Sirt1 immunohistochemistry in the hippocampus were consistent with the western blot of Sirt1 (Figure 7D).

## Discussion

Our present study demonstrated that, following a 21-day sleep restriction, the wild-type 12-month-old C57BL/6 female mice developed AD accompanied by cognitive deficiency, Aβ deposition and tau hyper-phosphorylation in the hippocampus, but these phenomenons can be reversed when treated with KD, indicating the prophylactic effect of KD on SD induced AD. Further mechanism revealed that this protective effect of KD is related with the inhibition of ferroptosis and the promotion of neuron repair *via* Sirt1/Nrf2 pathway in SD mice. In our knowledge, this is the first report on the prevention of SD-induced AD just by adjusting the diet to a mild KD.

Growing evidence has demonstrated that SD induces cognitive impairment which also is the clinical characteristic of AD (Zhao et al., 2017; Wang X. et al., 2021). The parameters of the MWM test showed that the escape latency was markedly increased, whereas the numbers of platform crossing and the times spent in the target phase in probe trials were obviously reduced in SD group mice. While KD improved chronic SD-induced cognitive deficiency, which is consistent with our previous study that KD prevented the cognitive deficiency induced by 21 days SD in younger mice (7-week-old) (Wang X. et al., 2022).

It has been reported that the sleep-wake cycle regulates the levels of Aβ (Holth et al., 2019). Aβ increases significantly following acute sleep deprivation and decreases following sleep recovery (Shokri-Kojori et al., 2018). Studies in AD transgenic mice have demonstrated that Aβ plaque in the hippocampus after a chronic sleep restriction (Rothman et al., 2013). What’s more, chronic SD promotes tau pathology spreading, which is also associated with the overproduction of Aβ (Holth et al., 2019). BACE1 is the major β-secretase and mediates the production of Aβ (Zhang and Song, 2013). In this work, the 21-day SD induced more Aβ deposition *via* up-regulating BACE1 and Aβ production, accompanied with increased p-tau in the hippocampus in the 12-month-old wild-type C57 BL/6 mice. The results were supported by the previous study demonstrating that Aβ plaque generated *via* up-regulating BACE1 in 9-month-old, adult and wild-type C57BL/6 mice after 60 days SD (Zhao et al., 2017). This phenomenon was reversed by KD treatment. In addition, it has been reported that sleep disturbances can impair Aβ clearance (Spinedi and Cardinali, 2019; Versele et al., 2020) found that ketone bodies (such as BHB) could promote Aβ clearance in a human *in vitro* blood-brain barrier model. Consideration of the elevated blood BHB in the present study (Supplementary Figure 1), it is reasonable to speculate that KD-induced the reduction of Aβ deposition and p-tau can be partially attributed to the promotion of Aβ clearance. These findings reinforced the causal link between SD and late-onset sporadic AD and proved a prophylactic intervention of moderate KD.

Growing evidence demonstrates that hippocampal damage is the main factor of SD-induced cognitive dysfunction (Zuo et al., 2020; Wang X. et al., 2021). Our present results showed that hippocampal neurons are characterized by pyknotic nuclei and vacuolated cytoplasm in SD group mice (Figure 4A). However, KD treatment alleviated this degeneration in SD-exposed mice. The effects were supported by our previous study showing that KD can attenuate chronic SD-induced hippocampal damage in young mice (7-weeks old) (Wang X. et al., 2022). DCX is a typical marker for adult neurogenesis and is close to cognitive abilities. The DCX diminished with aging, but is lower in AD patients than their peers (Moreno-Jiménez et al., 2019). BDNF is the most important neurotrophin involved in the growth, maintenance and survival of neurons, and a lower level of BDNF is related to a poorer cognition (Xue et al., 2022). Studies have shown that BDNF can induce DCX expression, and promote neurogenesis and reparation of neurons (Vitaliano et al., 2022; Xue et al., 2022). We found that 21-day SD induced a reduction in BDNF expression, as well as in DCX from the evidence of western blot and immunohistochemistry, which is in agreement with a previous study (Looti Bashiyan et al., 2021). These above indicate that SD suppressed neuronal repair and then enhanced hippocampal damage and cognitive deficiency. Notably, we found that KD upregulated the expression of BDNF and DCX, which suggested that the protective effect of KD on SD-induced hippocampal damage can be partly attributed to the promotion of repair in SD-exposed mice.

Emerging evidence indicated that SD-induced cognitive deficiency can be improved by Fer-1, a specific inhibitor of ferroptosis in mice, indicating ferroptosis involved in the mechanism of SD-induced cognitive deficiency (Wang X. et al., 2021, 2022). In addition, our previous study found that KD could improve chronic SD-induced hippocampal damage by inhibiting ferroptosis in younger mice (7-week-old). Accordingly, we investigated the effect of KD on ferroptosis-related indicators, including iron deposition, and lipid peroxides accumulation (Li et al., 2020). Improved Perls staining revealed that obviously intracellular iron accumulation in the hippocampus in the SD group mice in our present study. The results were supported by previous studies showing that an increase in intracellular iron in SD exposed mice (Wang X. et al., 2021, 2022). Similar to our previous study, the up-regulation of intracellular iron was reversed by KD treatment (Wang X. et al., 2022). What’s more, it has been demonstrated that iron accumulation can worsen senile plaque deposition and promote tau phosphorylation (Wang F. et al., 2022). More β-secretase is activated in the presence of excessive iron and then accelerating the Aβ production and amyloid deposition (Ayton et al., 2020; Gleason and Bush, 2021). In addition, Ayton and colleagues conducted a research demonstrating an association between iron accumulation and steeper rate of cognitive decline in subjects displaying significant amyloid plaques and tau tangles (Spotorno et al., 2020). Hence, the prophylactic effects of KD on SD-induced AD can be partially attributed to its inhibition of iron overload in the hippocampus.

Iron is uptake by TFR1 (Fe^3+^) or DMT1 (Fe^2+^) and can be temporarily stored in the ferritin in the cytoplasm after being oxidated by FTH1 to prevent its toxicity or exported by the membrane protein FPN1 to maintain intracellular iron homeostasis. Neuron maintains intracellular iron homeostasis by modulating the expression of above iron transporter proteins *via* the IRP/IRE (iron-responsive element) system (Jing et al., 2021). IRPs regulate the iron homeostasis by binding to IRE of aforementioned iron transporter proteins’ mRNAs dependent on cellular iron status (Vashisht et al., 2009). IRPs increase intracellular active iron *via* upregulating TfR1, downregulating FTH1 and FPN1. However, abnormal increase of IRPs can lead to the intracellular iron accumulation and thereby promoting ferroptosis. Animals with targeted deletions of IRP1 and IRP2 have demonstrated that IRP2 serves as the primary physiologic iron sensor, while IRP1 competes with IRP2 in regulating cellular iron homeostasis in response to ROS (Meyron-Holtz et al., 2004). We found SD elevated the expression of IRP1 rather than IRP2, thereby increasing TfR1 and DMT1, decreasing FTH1 and FPN1, leading to intracellular iron increase and deposition in the hippocampus. Nevertheless, KD successfully suppressed the up-regulation of IRP1, subsequently modified SD-induced abnormality of iron transporters with diminished iron aggregation. These results revealed that KD prevented chronic SD-induced AD by inhibiting hippocampal iron dyshomeostasis.

Excess active iron promotes ROS production *via* the Fenton reaction, leading to lipid peroxides accumulation and thereby triggering ferroptosis. It has been well-believed that lipid peroxide MDA accumulation could present the development of ferroptosis (Yuan et al., 2020). GPX4/xCT system is the main lipid peroxide removal system by reducing lipid peroxides to non-toxic lipid alcohols (Yan et al., 2021). Inhibition of GPX4 in neurons causes cognitive deficiency and neurodegeneration (Hambright et al., 2017). FSP1 is in parallel with GPX4/xCT system to suppress ferroptosis by reducing lipid peroxidation by producing a reduced form of CoQ10, a well-known potent antioxidant (Doll et al., 2019). Consistent with the elevated iron, an increase in MDA levels, a reduction in GSH level, as well as in FSP1, xCT and GPX4 expression were presented in the hippocampus of SD group mice. These effects were suppressed by KD treatment, suggesting that KD can promote lipid peroxide removal by elevating GPX4/xCT system and FSP1 to prevent ferroptosis. These above results verified our previous speculation, KD could prevent the occurrence of chronic SD-induced AD partly by inhibiting ferroptosis.

Based on the results aforementioned, we further explored the signaling pathways by which KD was exerting its effect on neuroprotection. As a class III histone deacetylase, Sirt1 plays a role in neuroprotection and longevity (Wang C. et al., 2021). Up-regulation of the expression of Sirt1 can improve cognitive function, and prevent the onset of AD (Cao et al., 2018). Zuo et al. (2020) demonstrated that H_2_S can prevent SD-induced hippocampal damage by up-regulating Sirt1 expression in the hippocampus, but this effect can be reversed by the Sirt1 inhibitor in rats. What’s more, it has been reported that melatonin alleviates short-term SD-induced memory loss in mice by suppressing hippocampal ferroptosis (Wang X. et al., 2021) and increasing hippocampal Sirt1 (Hinojosa-Godinez et al., 2019). These results indicate that Sirt1 may participate in the regulation of ferroptosis but less has been reported about the mechanism of Sirt1 on ferroptosis. Nrf2, a key regulator of the cellular antioxidant response, is a downstream target of Sirt1 and has been linked to neurodegenerative disease treatment and ferroptosis regulation. Activation of Nrf2 could against chronic SD-induced memory impairment in rats (Tang et al., 2020). Moreover, a recent study using the EX527 (an inhibitor of SIRT1) proved the anti-ferroptosis effect of Sirt1/Nrf2 pathway in a sepsis mice model (Qiongyue et al., 2022). A recent study demonstrated that Nrf2 induction could prevent ferroptosis in Friedreich’s Ataxia (La Rosa et al., 2021). Currently, it has been proved that Nrf2 not only contributes to mitigation of lipid peroxidation by activating GPX4/xCT axis (Yuan et al., 2021) but also modulates celluar iron homeostasis by increasing the expression of iron transport proteins FTH1 and FPN1 (Kerins and Ooi, 2018). In agreement with our previous study (Wang X. et al., 2022), we found that KD up-regulated the expression of Sirt1 and Nrf2 in SD exposed mice. In addition, Sirt1-Nrf2 is also associated with the neurogenesis by up-regulate the expression of BDNF and DCX (Namgyal et al., 2020; Suwannakot et al., 2021). These above results prompted us to conclude that KD prevents chronic SD-induced AD *via* Sirt1/Nrf2 signaling pathway.

In summary, our present study first demonstrated the prophylactic effect of KD on SD-induced AD in wild-type mice models *via* activating the Sirt1-Nrf2 pathway in the hippocampus. Our findings may provide new perspectives on the dietary treatment of AD.

## Data availability statement

The original contributions presented in the study are included in the article/Supplementary material, further inquiries can be directed to the corresponding author.

## Ethics statement

Our experimental protocol was approved by the Research and Ethics Committee of Liaocheng University.

## Author contributions

MW and ZW: conception and design of research. YY, XW, and AX: performed the experiments. YY, XW, and MW: analyzed the data. MW, YY, JH, and ZW: interpretation of the results of experiments. YY and XW: prepared the figures. YY and MW: drafted the manuscript. All authors read and approved the final manuscript.

## Supplementary material

Supplementary Figure 1 Effects of ketogenic diet (KD) on blood β-hydroxybutyrate (BHB) **(A)** and blood glucose **(B)** in sleep deprivation (SD) exposed mice (mean ± SEM, *n* = 10). Different letter indicates significantly different between each group (*p* < 0.05). **p* < 0.01, compared with Con; ^#^*p* < 0.01, compared with SD.
