# Supplementary material for: Ketogenic diet prevents chronic sleep deprivation-induced Alzheimer’s disease by inhibiting iron dyshomeostasis and promoting repair via Sirt1/Nrf2 pathway
Source: Front Aging Neurosci. 2022 Sep 1;14:998292. doi: 10.3389/fnagi.2022.998292 (PMC9475074; doi:10.3389/fnagi.2022.998292)
Supplement: Supplementary Figure 1 — Effects of ketogenic diet (KD) on blood β-hydroxybutyrate (BHB) (A) and blood glucose (B) in sleep deprivation (SD) exposed mice (mean ± SEM, n = 10). Different letter indicates significantly different between each group (p < 0.05). *p < 0.01, compared with Con; #p < 0.01, compared with SD. [file Data_Sheet_1.docx]

**Supplementary Table 1.** Ingredient compositions of experimental diets

| **Ingredient(g/kg)** | **AIN93M** | **KD** |
| --- | --- | --- |
| Corn starch | 465.7 | 300 |
| Dextrin | 155 | - |
| Sucrose | 100 | - |
| Casein | 140 | 140 |
| Powdered cellulose | 50 | 50 |
| Soybean oil | 40 | 460 |
| Mineral mix | 35 | 35 |
| Vitamin mix | 10 | 10 |
| Choline bitartrate | 2.5 | 2.5 |
| L-Cystine | 1.8 | 1.8 |
| t-buthlhydroquinone | 0.008 | 0.008 |
| Protein(% g) | 12.36 | 12.36 |
| Carbohydrate(% g)  Fat(% g) | 72.7  4.0 | 31.7  46.0 |
| Protein(kcal/g) | 0.62 | 0.50 |
| Carbohydrate(kcal/g)  Fat(kcal/g) | 2.62  0.36 | 1.27  4.14 |
|  |  |  |
| ^a^Ketogenic ratio | 0.05 | 1.05 |

**
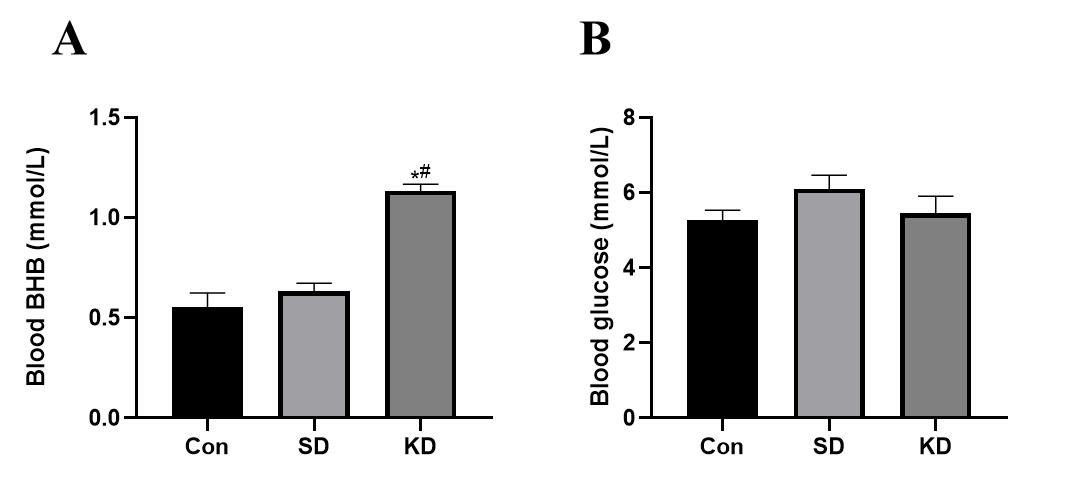
**

**Supplementary Figure 1.** Effects of KD on blood BHB (A) and blood glucose (B) in SD exposed mice (mean ± SEM, n=10). Different letter indicates significantly different between each group (*p* < 0.05).**p* <0.01, compared with Con; ^#^*p* <0.01, compared with SD.
